# A possible association between fructose consumption and pulmonary emphysema

**DOI:** 10.1038/s41598-019-45594-1

**Published:** 2019-06-27

**Authors:** Camila Liyoko Suehiro, Alessandra Choqueta de Toledo-Arruda, Rodolfo de Paula Vieira, Francine Maria de Almeida, Clarice Rosa Olivo, Milton de Arruda Martins, Chin Jia Lin

**Affiliations:** 1 Laboratory of Molecular Pathology (LIM-22), Department of Pathology, School of Medicine, University of Sao Paulo, Sao Paulo, Brazil; 2 Department of Medicine (LIM-20), School of Medicine, University of Sao Paulo, Sao Paulo, Brazil; 3 Brazilian Institute of Teaching and Research in Pulmonary and Exercise Immunology (IBEPIPE), School of Medical Sciences of Sao Jose dos Campos Humanitas and Universidade Brasil, Sao Jose dos Campos, Brazil

**Keywords:** Chronic obstructive pulmonary disease, Experimental models of disease

## Abstract

Chronic Obstructive Pulmonary Disease (COPD) is a syndrome that comprises several distinct and overlapping phenotypes. In addition to persistent airflow limitation and respiratory symptoms, COPD is also characterized by chronic systemic inflammation. Epidemiological studies have shown that dietary fibers, fruits and vegetables intake protects against the COPD development, while fructose-loading is associated with increased risk of asthma and chronic bronchitis. Since dietary factors might affect susceptibility to COPD by modulating oxidative stress and inflammatory responses, we evaluated how fructose feeding might affect the smoking-induced emphysema in mice. We found that chronic fructose intake induced destruction and remodeling of lung parenchyma and impairment of respiratory mechanics, which are associated with distinctive cytokine profiles in bronchoalveolar lavage fluid, blood plasma and skeletal muscle. The combined effects of chronic fructose intake and cigarette smoking on destruction of lung parenchyma are more pronounced than the effects of either alone. Excessive intake of fructose might directly cause pulmonary emphysema in mice rather than just altering its natural history by facilitating the installation of a low-grade systemic inflammatory milieu.

## Introduction

Chronic obstructive pulmonary disease (COPD) is a major cause of morbidity and mortality worldwide and it may become the third leading cause of death by 2030 according to estimates of World Health Organization^1^. COPD is a syndrome that comprises several distinct and overlapping phenotypes characterized by airflow limitation and chronic respiratory symptoms^2^. COPD arises from an interplay between exposure to environmental factors and host factors such as genetics, airway hyperresponsiveness and poor lung development^3^. The main morphological alterations underlying COPD are emphysema, obstructive bronchiolitis, and, in many cases, chronic bronchitis. The relative participation of each pathological feature varies between patients^2,3^. Thus, many COPD patients shows a combination, in different proportions, of both “pink puffer” (more emphysema, lung hyperinflation, and dyspnea) and “blue bloater” (more chronic bronchitis, expectoration, higher body mass index, higher frequency of cardiovascular comorbidities) classic phenotypes^2^.

COPD affects not only the lungs. Muscle dysfunction and cachexia is found in many COPD patients. Also, several morbidities such as malignant neoplasms, coronary artery disease, arrhythmias, hypertension, congestive heart disease, diabetes and metabolic syndrome occur in greater frequency among COPD patients than in general population^4,5^. For this reason, COPD can be considered as a systemic disease. The coexisting diseases impair the functional status, the quality of life and the survival of COPD patients^2,6^. Two hypotheses have been proposed to explain the relationship between COPD and its extra-pulmonary manifestations and comorbidities. The first one considers the systemic manifestations and comorbidities as the result of a systemic “spill-over” of the inflammatory and reparatory events occurring in the lungs of patients with COPD, while in the second hypothesis the COPD is viewed as a systemic inflammatory disease that involves multiple organs with the pulmonary manifestations being one of its facets^5^. Both hypotheses predict that attenuation of persistent inflammation will decrease the severity of COPD and its extra-pulmonary manifestations despite their significant divergence regarding the primary source of the inflammatory events.

Treating COPD patients with statins – a class of cholesterol lowering agents that has anti-inflammatory properties^7^ – reduces all-cause mortality and mortality due to cancer, COPD, or cardiovascular disease as well as the frequency of COPD exacerbations^8,9^. Interestingly, high consumption of dietary fiber or fruits and vegetables is associated with lower risk of COPD among individuals that has a (current or former) history of cigarette smoking^10,11^, while a diet rich in fiber-containing foods is associated with better lung function irrespectively of smoking status^12^. These findings support the notion that attenuation of inflammatory events by either pharmacological or non-pharmacological approach might be beneficial to COPD patients. They also suggest that dietary factors, due to their effects on oxidative stress and inflammation, might modulate a person’s susceptibility to the known risk factors for COPD.

A possible dietary factor that might affect the lung function and clinical course of COPD is the content of fructose in the food. High-fructose corn syrup (HFCS) is used as a sweetening agent for industrialized food and beverages in several countries^13,14^. Rodents fed with a high fructose diet develop hallmarks of metabolic syndrome (MS) like elevation of serum triglycerides, increased blood pressure, decreased insulin sensitivity with hyperinsulinemia and altered secretion of very low-density lipoprotein^15^. As MS is characterized by a persistent low-grade systemic inflammation an important question is whether and how the use of fructose as a food addictive might affect lung function. Moreover, retrospective studies have shown that ingestion of HFCS sweetened beverage or beverages that have excess free fructose (EFF, fructose to glucose ratio larger than 1:1) is associated to the risk of asthma and chronic bronchitis^16,17^. In the present work, we used a mouse model of cigarette-induced emphysema in the context of fructose-feeding to assess how chronic fructose intake interacts with exposition to cigarette smoke and affects the development of pulmonary emphysema. Our results suggest that chronic fructose ingestion may cause emphysema in mice independently of cigarette smoking.

## Results

### Body weight gain

**Figure 1:**
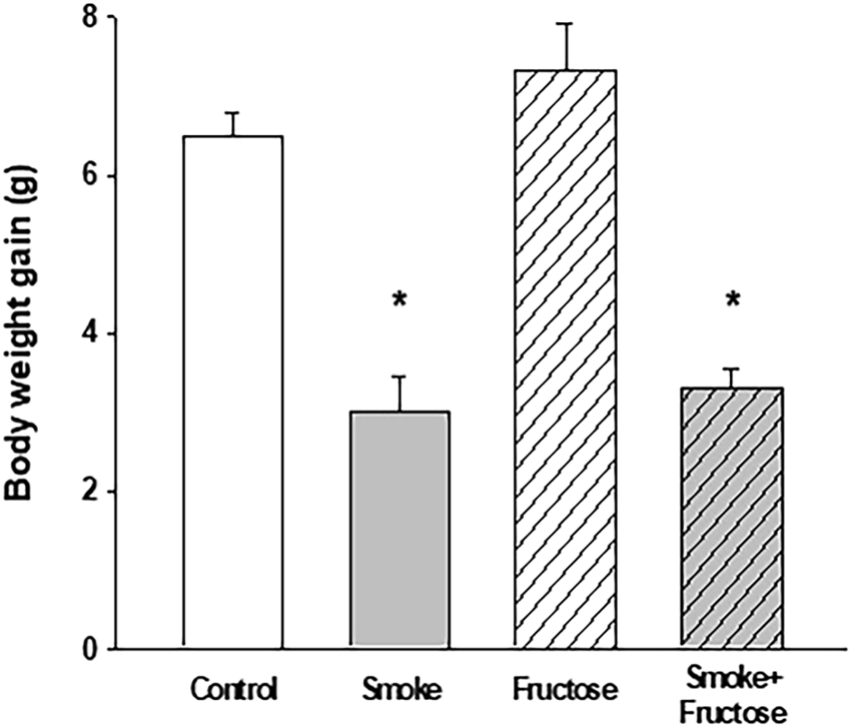
Body weight gain in grams of the mice after 12 weeks of the experimental protocols. Data are presented as mean and standard deviation. *p < 0.001 compared to the Control and Fructose groups.

After 12 weeks, animals from all the groups showed an increase in the body weight. Ingestion of fructose caused largest increase while smoking was associated with smallest increase in weight gain. Weight gain of animals allocated to Smoke and Smoke + Fructose groups was significantly lower than weight of the littermate assigned to, respectively, Control and Fructose groups (Fig. 1).

### Food, water and calorie intake

**Figure 2:**
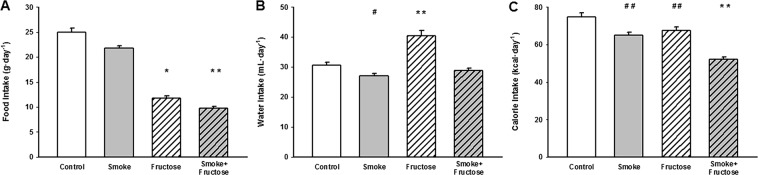
Food intake (**A**), water intake (**B**) and calorie intake (**C**). Data are presented as mean and standard deviation. *p < 0.001 compared to the Control and Fructose groups. **p < 0.001 compared to the other groups. ^#^*p* < 0.05 compared to the Control group. ^##^*p* < 0.01 compared to the Control group.

Exposure to either CS or fructose altered intake of food, fluid and calories. The Smoke group ingested less food than the Control group (Fig. 2A) while the Fructose group decreased food intake compared to either the Control or Smoke groups (Fig. 2A). The food intake was lower in the Smoke + Fructose group than either treatment alone (Fig. 2A). The water intake was higher in the Fructose group compared to the Control, Smoke or Smoke + Fructose groups (Fig. 2B). In contrast, the Smoke group ingested less water than the Control group (Fig. 2B). Both the Smoke and the Fructose groups showed a decreased calorie intake compared to the Control group (Fig. 2C). The calorie intake was lower in the Smoke + Fructose group than any treatment alone (Fig. 2C).

### Respiratory mechanics

**Figure 3:**
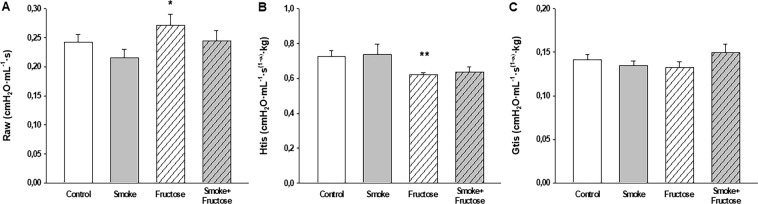
Airway resistance (Raw) (**A**), tissue elastance (Htis) (**B**) and tissue resistance (Gtis) (**C**) after 12 weeks of the experimental protocols. Data are presented as mean and standard deviation. *p < 0.05 compared to the Smoke group. **p < 0.05 compared to the Control group.

The Fructose group showed an increase in Raw compared to the Smoke group (Fig. 3A) and a decrease in Htis compared to the Control group (Fig. 3B). There were no significant differences in Gtis across the treatment groups (Fig. 3C).

### Cytological profile of BALF and lung tissue

**Figure 4:**
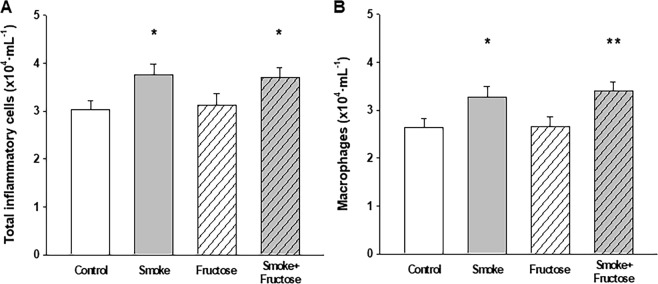
Total number of inflammatory cells (**A**) and macrophages (**B**) in the BALF. Data are presented as mean and standard deviation. *p < 0.05 compared to the Control group. **p < 0.05 compared to the Control and Fructose groups.

**Figure 5:**
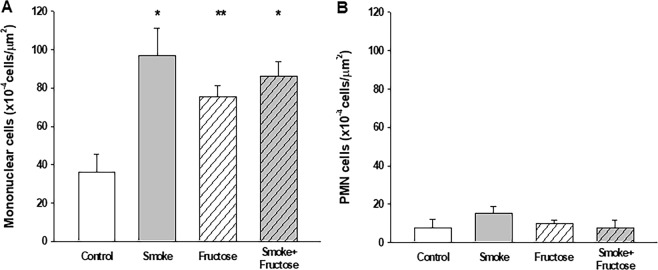
Mononuclear (**A**) and PMN (**B**) cells in the lung parenchyma. Data are presented as mean and standard deviation. *p ≤ 0.001 compared to the Control group. **p < 0.05 compared to the Control group.

Exposure to CS increased the number of inflammatory cells in BALF. Total number of inflammatory cells was increased in both Smoke and Smoke + Fructose groups compared to the Control group (Fig. 4A). This increase was due the macrophages, which was increased in the Smoke group and in the Smoke + Fructose group (Fig. 4B). There were no significant differences in the number of neutrophils or lymphocytes in BALF across the groups (data not shown). Exposure to fructose did not alter the number of inflammatory cells in BALF. Exposure to either CS or fructose increased the number of mononuclear cells in lung parenchyma (Fig. 5A). There were no significant differences in the number of PMN cells in lung parenchyma among groups (Fig. 5B).

### Lung histology

**Figure 6:**
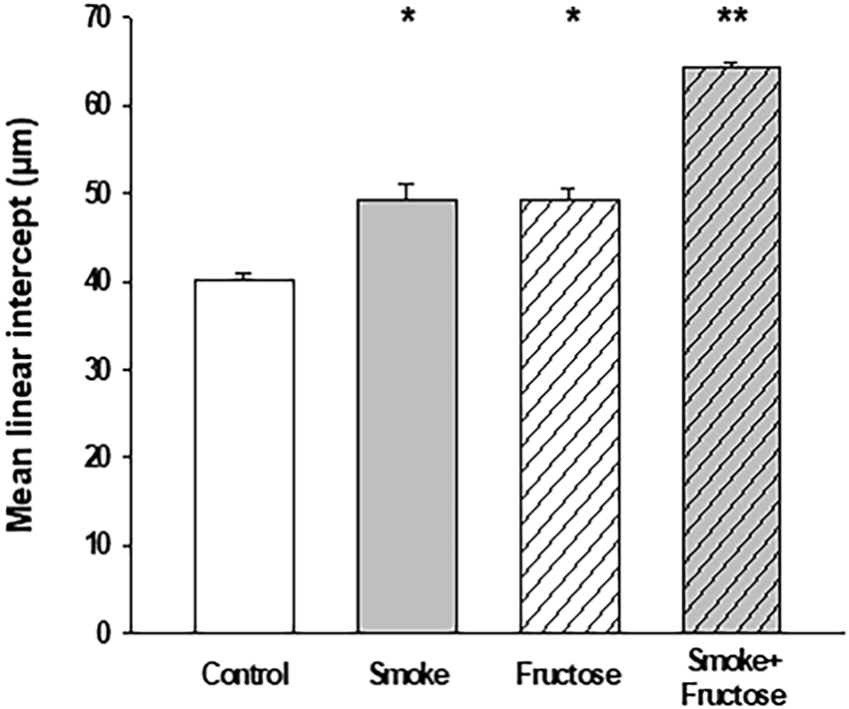
Mean linear intercept (Lm) after 12 weeks of the experimental protocols. Data are presented as mean and standard deviation. *p < 0.001 compared to the Control group. **p < 0.001 compared to the other groups.

**Figure 7:**
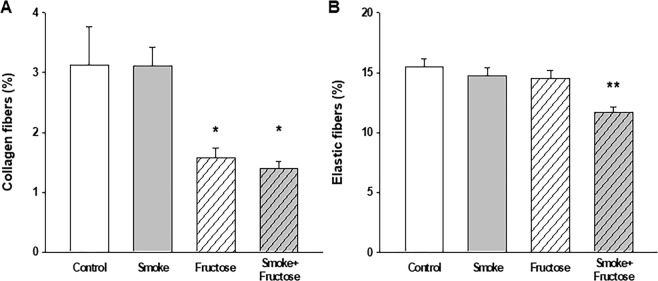
Percentage of collagen (**A**) and elastic (**B**) fibers in the lung parenchyma. Data are presented as mean and standard deviation. *p < 0.01 compared to the Control and Smoke groups. **p < 0.01 compared to the other groups.

Exposure to either CS or fructose resulted in an increase in the Lm. Both the Smoke and the Fructose groups showed an increase in the Lm compared to the Control group (Fig. 6). Combination of CS and fructose increased even further the Lm (Fig. 6). Exposure to fructose decreased the content of collagen fibers in the lungs. Both the Fructose and Smoke + Fructose groups showed a decrease in the content of collagen fibers compared to the Control and Smoke groups (Fig. 7A). The Smoke + Fructose group also showed a decrease in the content of elastic fibers in the lungs (Fig. 7B).

### Cytokines analyses in the BALF

The Smoke and Fructose groups showed an increase in BALF leptin levels compared to the Control group (Table 1). Fructose exposure, alone or in combination with the CS, increased the adiponectin levels (Table 1). The Smoke + Fructose group showed an increase in IL-1ra levels compared to the Control group (Table 1). There were no statistically significant differences in the levels of IL-10, IL-6, IL-1β and TNF-α in the BALF among groups (Table 1).

**Table 1:** Cytokines levels in the BALF, blood plasma and muscle homogenate.

	Control	Smoke	Fructose	Smoke + Fructose
***BALF***				
IL-10	15.131 ± 1.562	13.570 ± 1.831	18.220 ± 1.638	16.492 ± 1.638
IL-6	14.492 ± 3,178	17.271 ± 3.553	15.683 ± 3.178	12.542 ± 3.178
IL-1β	122.571 ± 9.211	108,786 ± 10,800	119,868 ± 10,183	123,419 ± 9,660
IL-1ra	12,635 ± 0,766	14,374 ± 0,898	14,416 ± 0,803	16,524 ± 0,847^a^
TNF-α	46,075 ± 2,779	50,683 ± 3,483	50,948 ± 3,072	52,908 ± 2,914
Leptin	133,994 ± 16,463	190,658 ± 20,637^a^	184,198 ± 17,266^a^	148,479 ± 18,200
Adiponectin	35,752 ± 55,555	32,683 ± 65,144	722,633 ± 61,418^j^	375,217 ± 58,267^b,f^
***Blood plasma***				
IL-10	33,949 ± 28,212	71,319 ± 26,119	149,976 ± 24,432^b^	197,309 ± 26,119^c,e^
IL-6	21,272 ± 6,483	10,365 ± 6,002	31,726 ± 5,614^d^	32,079 ± 6,002^d^
IL-1β	295,596 ± 30,458	270,963 ± 28,198	397,272 ± 26,377^i^	310,096 ± 30,458
IL-1ra	274,438 ± 25,455	261,376 ± 23,567	190,470 ± 22,045^a^	218,477 ± 23,567
TNF-α	24,409 ± 3,823	21,996 ± 3,539	24,230 ± 3,310	25,897 ± 3,539
Leptin	2149,409 ± 554,284	1596,220 ± 554,284	2615,494 ± 554,284	373,860 ± 592,555^c,g^
Adiponectin	2013,072 ± 714,101	2262,935 ± 661,129	1819,221 ± 618,430	3388,850 ± 661,129
***Muscle homogenate***				
IL-10	30,221 ± 11,010	29,343 ± 11,010	72,362 ± 11,010^a,e^	99,504 ± 11,771^b,f^
IL-6	19,802 ± 4,106	22,779 ± 4,106	34,775 ± 4,106^a^	38,319 ± 4,389^b,d^
IL-1β	136,936 ± 7,387	133,055 ± 7,387	175,210 ± 7,387^c,d^	182,130 ± 7,897^c,f^
IL-1ra	257,697 ± 6,969	261,974 ± 6,969	252,811 ± 6,969	261,289 ± 7,451
TNF-α	20,020 ± 1,403	17,836 ± 1,403	23,786 ± 1,403^d^	27,888 ± 1,499 ^b, f^
Leptin	1715,085 ± 700,797	1346,756 ± 700,797	6482,549 ± 749,183^c,f^	4747,476 ± 749,183^a,e^
Adiponectin	91,846 ± 84,009	82,094 ± 84,009	1148,719 ± 84,009^c,f,h^	820,123 ± 89,809^c,f^

Data are presented as mean and standard deviation. ^a^*p* < 0.05 compared to the Control group; ^b^*p* < 0.01 com*p*ared to the Control group; ^c^*p* ≤ 0.001 compared to the Control group; ^d^*p* < 0.05 compared to the Smoke group; ^e^*p* < 0.01 compared to the Smoke group; ^f^*p* < 0.001 compared to the Smoke group; ^g^*p* ≤ 0.01 compared to the Fructose group; ^h^*p* < 0.05 com*p*ared to the Smoke + Fructose group; ^i^*p* < 0.05 compared to the other groups; ^j^*p* < 0.001 compared to the other groups.

### Cytokines analyses in the blood plasma

The plasma leptin levels in Smoke + Fructose group was decreased compared to the Control and Fructose groups (Table 1). Ingestion of fructose, with CS exposure or not, was associated with increased IL-10 levels (Table 1). Fructose exposure also increased the IL-6 levels (Table 1). The Fructose group showed an increase in IL-1β levels compared to all other groups (Table 1) and a decrease in IL-1ra levels compared to the Control group (Table 1). There were no statistically significant differences in the levels of adiponectin and TNF-α in the blood plasma among groups (Table 1).

### Cytokines analyses in the muscle homogenate

Exposure to fructose increased leptin and adiponectin levels in muscle homogenate regardless of CS exposure (Table 1). Levels of IL-10 were increased in both Fructose and Smoke + Fructose groups compared to the Control and Smoke groups (Table 1). Fructose feeding, independently of CS exposure, elevated the levels of IL-6 in muscle homogenate (Table 1). Both Fructose and Smoke + Fructose groups showed an increase in IL-1β levels compared to the Control or the Smoke groups (Table 1). The Fructose group showed an increase in TNF-α levels compared to the Smoke group (Table 1). The Smoke + Fructose group showed an increase in TNF-α levels compared to the Control and Smoke groups (Table 1). There were no statistically significant differences in the levels of IL-1ra in the muscle homogenate among groups (Table 1).

## Discussion

In the present study, we showed that chronic fructose loading promotes destruction and remodeling of lung parenchyma and impaired respiratory mechanics. Such alterations in the structure and function of lung parenchyma are associated with distinctive cytokine profiles in BALF, blood plasma and skeletal muscle. Furthermore, the combined effects of chronic fructose intake and CS on destruction of lung parenchyma are more pronounced than the effects of either treatment alone. These results suggest that excessive intake of fructose might be a cause of alveolar destruction and pulmonary emphysema in C57BL/6 mice rather than just playing an accessory role of creating a systemic inflammatory milieu, which might alter the natural history of emphysema induced by inhaled noxious stimuli. This is an unexpected finding given the previously reported relationship between excessive body weight or adiposity, insulin resistance and airflow limitation^18–20^. Our results complement reports from other authors^16,17,21,22^ and suggest that, in addition to airway hyperresponsiveness and chronic bronchitis, alveolar remodeling and emphysema might also be a deleterious consequence of chronic fructose exposure. The accumulating evidences of negative health effects of fructose, HFCS, and certain fruit drinks illustrate the importance of a deeper understanding on the relationship between dietary habits and noncommunicable disease.

The lung injury presented by our fructose-loaded animals bears similarities with the emphysema caused by CS exposure as both are characterized by increased Lm and Htis. However, unlike the CS-exposed mice in this study, our fructose-exposed mice present increased airway resistance and decreased content of collagen fiber in their alveolar septa. Previous work from our group have shown that C57BL/6 mice exposed to CS develop pulmonary emphysema without showing significant alterations in airway resistance^23^. Also, a study using specimens of human lung tissue^24^ and our group’s observations using mouse models of experimentally induced emphysema^23,25^ have shown that CS-induced emphysema is accompanied by an increase in the collagen content in alveolar wall. These specific features of fructose-associated pulmonary lesion suggest that fructose load and CS exposure rely on different pathophysiological mechanisms to produce alveolar lesion.

Besides the difference in airway resistance and collagen content of alveolar septa, our fructose-loaded mice present a distinctive profile of leukocytes in BALF and lung parenchyma when compared to CS-exposed littermates. Exposure to CS increases the number of inflammatory cells in BALF due to increased number of macrophages with no significant change in the number of neutrophils and lymphocytes. In contrast, fructose loading does not alter the number or composition of cells in BALF. It is noteworthy that both CS exposure and fructose loading causes an increase in the number of mononuclear cells that infiltrate the lung parenchyma. The difference between CS and fructose exposure in cytological pattern of BALF might be explained by differences in how CS and fructose exposure cause the inflammation in lung parenchyma. In the former the irritation caused by CS on airway epithelium and alveolar surface recruits inflammatory cells to lung parenchyma and alveolar space, while in the latter fructose ingestion causes inflammatory response elsewhere which is propagated to lung parenchyma causing the accumulation of mononuclear cells only in this tissue.

Except for an increased concentration of leptin in BALF mice exposed to CS have no significant change in cytokine profile. In contrast, the fructose-fed mice exhibit multiple alterations in cytokine profile. The increased concentrations of IL-10, IL-6, IL-1β and TNFα in muscle homogenate are probably markers of skeletal muscle inflammation caused by chronic fructose overload while the elevated accumulation of both leptin and adiponectin in this material can be viewed as a protective response to limit the deleterious effects of fructose-induced metabolic overload^26^. Similarly, elevated concentration of IL-10, IL-6, IL-1β and decreased concentration of IL-1ra in blood plasma might be markers of a fructose-induced systemic inflammatory state. Finally, high concentration of leptin and adiponectin in BALF of fructose-exposed mice might have a connection with accumulation of collagen in the alveolar septa as both have been reported to modulate the remodeling of extracellular matrix in cardiovascular tissues^27,28^.

One possible explanation for the link between fructose consumption and pulmonary emphysema is the involvement of the lungs by an inflammatory response initiated in an extra-pulmonary site by chronic fructose consumption. The liver is a natural candidate for the initiation site of fructose-triggered inflammatory state, since it is the major site for metabolism of orally ingested fructose^29^ and ingestion of fructose has been reported to cause hepatic stress response, activation of c-Jun N-terminal kinases and inflammatory response in rodents^30^. A competing explanation is the “intestinal enFruAGE hypothesis”^31,32^. According to this hypothesis, EFF in the ingested food or beverages promotes, due to fructose malabsorption, the formation of advanced glycation endproducts in the intestinal lumen which engage receptor for advanced glycation endproducts (RAGE) after being translocated into circulation. Inflammation elicited by activation of RAGE would be the mechanism underlying the association between the ingestion of foods with EFF and chronic inflammatory diseases. In support of this hypothesis, RAGE is highly expressed in lung and has been shown to play a central role in the pathogenesis of asthma^33,34^. Secondly, regular consumption of EFF beverages has been reported to be associated with childhood or adult asthma^17,21^, chronic bronchitis^16^, and arthritis^35^ whereas beverages that do not have EFF show no such associations. However, a study comparing mice fed with high-fat diet and mice fed with high-fructose diet seems to challenge the “intestinal enFruAGE hypothesis”, as airway hyperresponsiveness was observed in either model of MS, irrespectively of fructose ingestion^22^. Furthermore, fructose malabsorption - a phenomenon that is central to the “intestinal enFruAGE hypothesis” - may not occur in rodents as fructose malabsorption protects against accumulation of fat in liver^36^ and it is known that both mouse and rat develop liver steatosis after receiving fructose in drinking water for 8 weeks^37^. Finally, none of our fructose-fed mice displayed enlarged intestines – a gross morphological alteration presented by mice that have fructose malabsorption^38^ – on autopsy. Thus, it is unlikely that significant fructose malabsorption had occurred in our fructose-exposed mice.

Our study has some limitations; we did not evaluate the effects of fructose load and CS exposure on the oxidant-antioxidant balance and protease-antiprotease balance on the emphysema development. Therefore, our results do not allow us to assess the involvement of these mechanisms in the destruction of alveoli caused by fructose exposure. In addition, we did not assess how different dosages of fructose might alter the severity of lung emphysema and missed an opportunity to test the “intestinal enFruAGE hypothesis”. Furthermore, we cannot conclude whether the alveolar destruction is a specific effect of fructose ingestion or it can be produced by excessive loading of other monosaccharides as we did not include groups of animals to address this question. A sucrose consuming group should have also been included to assess similarities/differences in response to fructose fed group, since, according to “intestinal enFruAGE hypothesis”, the deleterious effects of fructose on the respiratory system would be related to the high fructose to glucose ratios, and not with the amount of ingested fructose. It would have been of interest if we included a group that mimic fructose malabsorption, i.e. GLUT5 knockout group. Future studies should be performed to clarify these points.

In summary, we have shown that chronic fructose intake promotes destruction and remodeling of lung parenchyma and impairment of respiratory mechanics, which is associated with distinctive cytokine profiles in BALF, blood plasma and skeletal muscle. The direct connection between chronic intake of a macronutrient and chronic respiratory disease reported in this study illustrates the importance of a deeper understanding on the relationship between dietary habits and noncommunicable disease.

## Methods

### Animal handling and experimental protocol

Six to eight weeks old male C57BL/6J mice were provided by the University of Sao Paulo School of Medicine’s Animal Facility. The animals were randomly allocated into one of four experimental groups (n = 8–10 per group): Control; Smoke; Fructose and Smoke + Fructose and treated accordingly for 12 weeks. The animals were kept in cages with four to five animals under a 12-h light/dark cycle and were given *ad libitum* access to food and water. Standard chow (2990 kcal/kg) was given as a solid diet. The mice assigned to fructose treatment were given a 20% (wt/vol) fructose (Lowçucar®, PR, Brazil) as drinking solution. Fructose or cigarette smoke (CS) exposure began on the same day.

Body weight (g) was recorded for each mouse once a month. Food (g·day^−1^) and water intake (ml·day^−1^) were assessed weekly. Total calorie intake (kcal·day^−1^) was calculated from the weight of food and the volume of fluid ingested.

This study was approved by the Ethics Committee of the School of Medicine of the University of Sao Paulo (Sao Paulo, Brazil, protocol 001/14) and all animal handling and experiments were performed according to the procedures approved at our institution.

### Cigarette smoke exposure

Animals assigned to Smoke or Smoke + Fructose groups were exposed to CS for 30 minutes, twice·daily, 5 days·a week, for 12 weeks by using a custom-made smoking machine following previously published protocol^23^. The animals were exposed to 11 (±1) commercially filtered cigarettes (0.8 mg of nicotine, 10 mg of CO and 10 mg of tar per cigarette) per session in this study. Control and Fructose groups were exposed to room air.

### Respiratory mechanics

Twenty-four hours after the end of the exposure protocols, animals were anaesthetized (50 mg·kg^−1^ intraperitoneal thiopental) tracheostomized and mechanically ventilated (FlexiVent, Scireq, Montreal, QC, Canada). Breathing efforts were abolished by pancuronium (0.2 mg·kg^−1^ intraperitoneal). The forced oscillatory technique and a constant phase model were used to obtain airway resistance (Raw), tissue damping (Gtis) and tissue elastance (Htis) parameters^39^. The values of Gtis and Htis were normalized by body weight^40^.

### Blood plasma collection

The animals were exsanguinated via puncture of the abdominal aorta immediately after respiratory mechanics measurements. The blood was collected and centrifuged at 3000 g for 10 minutes at 5 °C. The plasma was stored at −80 °C for further analysis.

### Bronchoalveolar lavage fluid

Bronchoalveolar lavage fluid (BALF) samples were collected after washing the lungs with 3 × 0.5 ml of sterile 0.9% saline and were centrifuged at 900 g for 10 minutes at 5 °C. The supernatant was collected and stored at −80 °C for further analysis and the cell pellet was resuspended in 300 μL of 0.9% saline. The cells were counted by using a Neubauer haemocytometer chamber (Carl Roth, Karlsruhe, Germany) and cytological examination was performed by examining 300 cells per slide under 1000X magnification after preparing BALF samples in cytocentrifuge slides and stained with Diff Quick (Medion Diagnostics, Dündingen, Switzerland)^41^.

### Lung histology

For histological studies, lungs were removed and fixed at a constant pressure of 20 cm H_2_O for 24 h. Five-micrometers thick sections of lung tissue were stained with haematoxylin and eosin to evaluate the density of the polymorphonuclear (PMN) and mononuclear cells in the lung parenchyma^42^ and to measure the mean linear intercept (Lm), as described previously^43^. The content of collagen and elastic fibers were evaluated after staining, respectively, with Sirius-Red or Oxidate Weigert’s Resorcin-Fuchsin. The areas that were positive to the stains of collagen or elastic fibers was measured as described previously and expressed as a percentage of the total parenchyma area^44^.

### Enzyme-linked immunosorbent assay (ELISA)

Tumour necrosis factor alpha (TNF-α), interleukin (IL)-6), IL-10, IL-1β, IL-1ra, leptin and adiponectin were assayed in BALF supernatant, blood plasma and skeletal muscle (*quadriceps femori*) homogenate using ELISA kits (eBioscience, San Diego, CA, USA), according to the manufacturer’s instructions as described previously. The intensity of colorimetric reaction was measured by absorbance at 450 nm and the results are expressed in pg/mL^45^.

### Statistical analysis

Two-way analysis of variance was used to assess the effects of CS or fructose and Method of Holm-Sidak was used for pairwise multiple comparison. The analyses were performed using Sigma Stat 11 software (Systat Software, Inc., San Jose, CA, USA). A value of *p* < 0.05 was considered significant.

## Data Availability

All data generated or analyzed during this study are included in this published article.
