# Author Correction: A possible association between fructose consumption and pulmonary emphysema

**DOI:** 10.1038/s41598-020-69892-1

**Published:** 2020-09-25

**Authors:** Camila Liyoko Suehiro, Alessandra Choqueta de Toledo-Arruda, Rodolfo de Paula Vieira, Francine Maria de Almeida, Clarice Rosa Olivo, Milton de Arruda Martins, Chin Jia Lin

**Affiliations:** 1 Laboratory of Molecular Pathology (LIM-22), Department of Pathology, School of Medicine, University of Sao Paulo, Sao Paulo, Brazil; 2 Department of Medicine (LIM-20), School of Medicine, University of Sao Paulo, Sao Paulo, Brazil; 3 Federal University of Sao Paulo (UNIFESP), Post-Graduation Program in Sciences of Human Movement and Rehabilitation, Santos - SP, Brazil; 4 Universidade Brasil, Post-Graduation Program in Bioengineering and in Biomedical Engineering, Sao Paulo - SP, Brazil; 5 Brazilian Institute of Teaching and Research in Pulmonary and Exercise Immunology (LABPEI), Sao Jose dos Campos - SP, Brazil; 6 Anhembi Morumbi University, School of Medicine, Sao Jose dos Campos - SP, Brazil

Correction to: *Scientific Reports* 10.1038/s41598-019-45594-1, published online 27 June 2019

The original version of this Article contained errors.

The original Affiliation 3 was incorrectly given as ‘Brazilian Institute of Teaching and Research in Pulmonary and Exercise Immunology (IBEPIPE), School of Medical Sciences of Sao Jose dos Campos Humanitas and Universidade Brasil, Sao Jose dos Campos, Brazil’, the correct version is now listed as Affiliation 5.

Additionally, affiliations were omitted for the author Rodolfo de Paula Vieira, which are now listed as Affiliations 3, 4 and 6.

The correct affiliations are listed below:

Affiliation 1:

Laboratory of Molecular Pathology (LIM-22), Department of Pathology, School of Medicine, University of Sao Paulo, Sao Paulo, Brazil.

Affiliation 2:

Department of Medicine (LIM-20), School of Medicine, University of Sao Paulo, Sao Paulo, Brazil.

Affiliation 3:

Federal University of Sao Paulo (UNIFESP), Post-Graduation Program in Sciences of Human Movement and Rehabilitation, Santos - SP, Brazil.

Affiliation 4:

Universidade Brasil, Post-graduation Program in Bioengineering and in Biomedical Engineering, Sao Paulo - SP, Brazil.

Affiliation 5:

Brazilian Institute of Teaching and Research in Pulmonary and Exercise Immunology (LABPEI), Sao Jose dos Campos - SP, Brazil.

Affiliation 6:

Anhembi Morumbi University, School of Medicine, Sao Jose dos Campos - SP, Brazil.

These errors have now been corrected in the HTML and PDF versions of the Article.

